# Intestinal cellular localization of PCNA protein and CYP1A mRNA in Atlantic salmon *Salmo salar *L. exposed to a model toxicant

**DOI:** 10.1186/1472-6793-9-3

**Published:** 2009-03-23

**Authors:** Monica Sanden, Pål A Olsvik

**Affiliations:** 1 National Institute of Nutrition and Seafood Research (NIFES), N-5817 Bergen, Norway

## Abstract

**Background:**

The aim of the study was to examine the intestinal cellular localization of proliferating cell nuclear antigen (PCNA) and cytochrome P450 A1 (CYP1A) expression in Atlantic salmon *Salmo salar *L. exposed to a model toxicant. The stress response was induced by intraperitoneal injection of four salmon with a single dose (50 mg/kg) of the CYP1A inducer β-naphthoflavone (BNF) and intestinal tissue (mid and distal intestine; MI and DI) was sampled seven days later. Samples for histology and gene transcription analysis were collected from four exposed fish and four control fish. PCNA was assessed by immunohistochemistry, CYP1A mRNA was studied by *in situ *hybridization (ISH) and finally the transcription of five genes was quantified by real-time quantitative RT-PCR (real-time RT-PCR); two detoxifying genes (CYP1A and glutathione S-transferase; GST), a stress marker gene (heat shock protein 70; HSP70), PCNA and a gene marker of apoptosis (caspase 6A).

**Results:**

PCNA protein and CYP1A mRNA were successfully localized in the intestinal cells (MI) of both experimental groups. At the cellular level, BNF significantly lowered intestinal cell proliferation and increased the CYP1A mRNA levels compared to the control group. The real-time RT-PCR data, which showed an increased mRNA expression both in the MI and DI of 139- and 62-fold, respectively, confirmed the increased cellular CYP1A mRNA levels detected using ISH. HSP70 expression was also up-regulated in the exposed fish. The other examined genes did not show any differential regulation in the experimental fish group.

**Conclusion:**

This study showed that CYP1A mRNA had a specific intestinal cellular transcription pattern in Atlantic salmon exposed to BNF. At the cellular level CYP1A mRNA expression was always observed at or around the cell nucleus close to the basolateral cell membrane and at the tissue level CYP1A mRNA expression was most frequently observed in the basal and apex area of the intestinal folds. Taken together, a link between the intestinal detoxification system (CYP1A) and cell renewal system (PCNA) is indicated with these two processes being inversely correlated in BNF exposed fish.

## Background

The intestinal tract is a complex organ with functions related to digestion, absorption, endocrine regulation of digestion and metabolism, water and electrolyte balance and immunity [1]. As the aquatic environment is continuously contaminated with foreign organic chemicals it is essential to know how these compounds affect molecular and cellular mechanisms in the intestinal tract. Under normal homeostatic circumstances, new cells that are formed at the basal area of the intestinal folds and during cell differentiation cells migrate to the apex area where they are shred off during the process of apoptosis [2]. The interplay between cellular proliferation, differentiation and regulated cell death is essential for the maintenance of the intestinal tract [3].

In the fish intestine it has been demonstrated that homeostasis and structural features may be disturbed by dietary factors [4-8], dietary restriction [9-11] and exposure to xenobiotics [6,12,13]. Inhibition or stimulation of proliferation could be the first sign of abnormality in the intestinal tract and is often used as an early warning biomarker [5,14,15]. Cell proliferation can be investigated by immunohistochemical staining of the proliferating cell nuclear antigen (PCNA) [16] and by real-time RT-PCR analysis of PCNA transcription levels. PCNA is a 36 kD nuclear protein required for DNA synthesis and repair, and is closely associated with DNA polymerase in the S-phase of the cell cycle. Previous studies have described intestinal cell renewal in teleosts, showing that proliferating cells are found at the base of the intestinal fold [17,18]. However, it has also been found that a definite zone of proliferation in fish is difficult to identify [11,19].

Although the initialization of the apoptotic process varies widely between species, tissues and conditions, the intracellular apoptotic process is highly conserved. Caspases, which are the executioners of apoptosis, can be divided into two classes, the initiators and the effectors. The examined protein in the present study, caspase 6A, belongs to the effector caspases, and its expression is tightly regulated in the apoptotic pathway [20]

Components of the monooxygenase system (including CYP1A) are found along the whole length of the fish intestinal tract, but the highest activity is found in the proximal portions [21]. Along with CYP1A, heat shock protein 70 (HSP70) is one of the most studied proteins known to respond to a number of external stressors in fish [22-25]. HSP70 is an indicator of stress or exposure to toxicants [26] and evidence shows that it also protects cells from apoptosis [27].

In the present study β-naphthoflavone (BNF), an aryl hydrocarbon receptor (AhR) agonist and a known model toxicant, was intraperitoneal (i.p) injected into the experimental fish. The effects of BNF have been reported in several fish species and are well known [28-30]. The relationship between AhR, the cell renewal system [31] and several biotransformation enzymes, including cytochrome P450 1A (CYP1A) [25,32] and glutathione S-transferases (GSTs) [33] is well documented. However, little information is available on the cellular relationship and localization of the intestinal integrity parameter (PCNA protein) and the toxicology parameter (CYP1A mRNA) in a stressed intestine.

**Figure 1:**
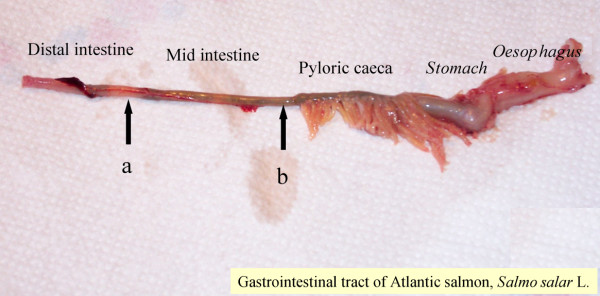
**Gastrointestinal tract of Atlantic salmon (≈ 100 grams) showing oesophagus, stomach, pyloric caeca, mid intestine (MI) and distal intestine (DI)**. A) Arrow showing where the DI samples were taken for gene expression B) Arrow showing where the MI samples were taken for gene expression, immunohistochemistry and *in situ *hybridization.

The aim of the study was to examine the cellular localization of CYP1A mRNA and PCNA protein in a stress-induced intestine. Tissues from the middle part of the intestine (MI) and the distal part of the intestine (DI) were sampled for analysis (Fig. 1). To confirm that the model toxicant successfully induced a stress reaction in the intestine, the expression of five genes encoding proteins involved in cellular response to stressors were examined; CYP1A, HSP70, GST, PCNA and caspase 6A by real-time RT-PCR. The histological parameters PCNA protein and CYP1A mRNA, were only examined in the MI since previous observations indicate a higher metabolic capacity in the proximal intestine of fish compared to the distal parts [10,11,18].

## Results

**Figure 2:**
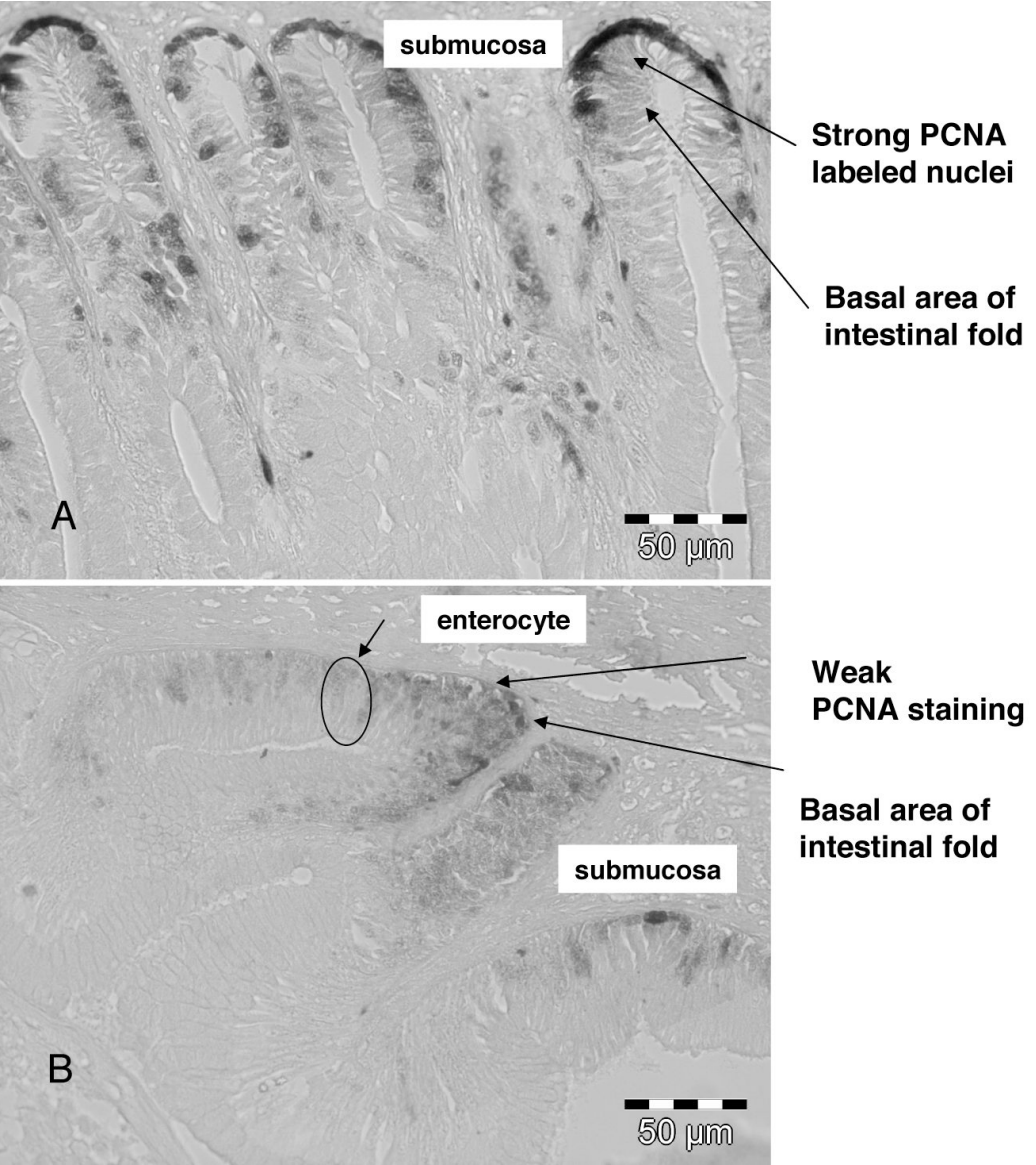
**Light photomicrographs of PCNA labelled nuclei in the mid intestine (MI) of Atlantic salmon **(A) In the control group, proliferating cells (PCNA) can be seen (strong black nuclear staining) in several cells in the basal area of the intestinal folds (B) In the treated group, proliferating cells (PCNA) can be seen (weak faint nuclear staining) only in a few cells in the basal area of the intestinal fold.

A single dose of β-naphthoflavone (BNF) significantly decreased the intestinal cell proliferation in the mid intestine (MI). Fish exposed to BNF had a significantly lower density (%) of PCNA-positive cells compared to fish in the control group (t-test: % PCNA, p < 0.03). Exposed fish had a PCNA index of 19.8 ± 4.5 (mean ± SD) compared to the PCNA index of 27.3 ± 4.1 (mean ± SD) in the control group (only for the MI). The relative quantification of PCNA positive cells confirmed the visual differences between the two experimental groups (Fig. 2). For both groups proliferating cells were observed in the basal area of the MI folds with only a few PCNA stained cells located in the cell differentiation zone. For quantification, only strongly stained cells located in the basal area 15 cells from the middle basal area were considered. Fig. 2A shows a representative section of cell proliferation in fish from the control group. Several cells in the basal area are strongly stained and assumed to be in the S-phase of the cell cycle. Fig. 2B shows a representative section of cell proliferation in fish from the exposed group and only weak PCNA staining can be seen. Only a few cells were strongly stained in the examined sections and generally the proliferative compartment length (PCL) (not measured) was smaller compared to the PCL in the exposed group. A brief description of intestinal PCL measurements can be found in Bakke-McKellep *et al*. [8].

Since the effect of BNF on CYP1A expression (see next paragraph) was much stronger in the MI samples compared to the distal intestine (DI) tissue, histological evaluations were performed only on this part of the intestinal tract.

**Figure 3:**
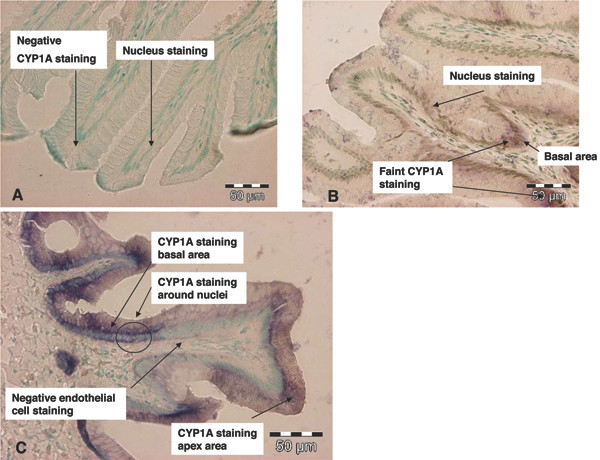
**Light photomicrographs of CYP1A mRNA labelled mid intestine (MI) of Atlantic salmon (A) Negative control (sense)**. No CYP1A staining and only nuclear staining can be seen (B) Control group (antisense). CYP1A staining with faint and low mRNA levels especially in the basal area of the intestinal folds (C) Exposed group (antisense). CYP1A staining with strong and high mRNA levels especially in the basal area and the apex area of the intestinal fold. Inside the cells CYP1A are located at and around the nucleus. In the photographs CYP1A mRNA levels can be seen with a dark colour and all cell nuclei with a green staining. All pictures are stained with the nuclear stain; methyl green.

Results of CYP1A mRNA transcription analysis by ISH in the MI can be seen in Fig. 3. In the exposed groups (Fig 3C) CYP1A mRNA was mainly localized in the basal area and apex area of the intestinal folds. Only weak staining could be seen in the cell differentiation zone of the MI fold. This expression pattern was the same in all fish exposed to BNF. CYP1A mRNA was mainly seen in one intestinal cell type, the enterocytes. Another interesting observation was the intracellular localization of the CYP1A transcript around the nucleus close to the basolateral membrane. CYP1A transcripts were only seen as weak and random staining in the cytoplasm close to the apical membrane. This feature was prominent both in the basal area and apex area of the MI fold. Results of CYP1A mRNA transcription in the control fish can be seen in Fig. 3B. In this group only weak signals could be seen and with no particular cellular or tissue expression pattern. Fig. 3A shows a representative example of a sense probe control.

**Figure 4:**
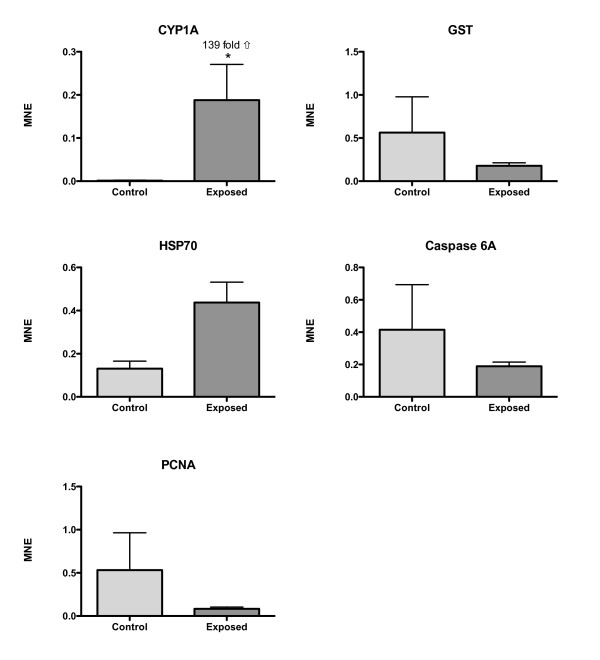
**Mean normalized expression (MNE) of CYP1A, GST, HSP70, PCNA and caspase 6A in the mid intestine (MI) of Atlantic salmon exposed to BNF**. An asterisk denotes a significant altered expression. Fold-change induction is shown in the figure. Mean ± SEM. n = 4.

**Figure 5:**
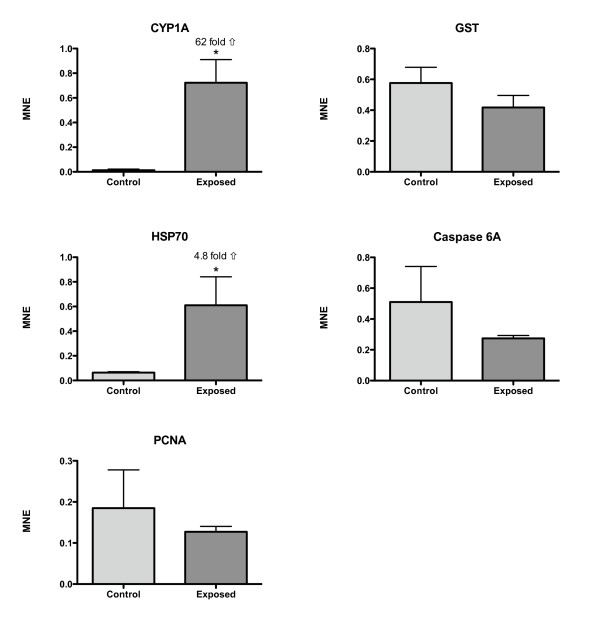
**Mean normalized expression (MNE) of CYP1A, GST, HSP70, PCNA and caspase 6A in the distal intestine (DI) of Atlantic salmon exposed to BNF**. An asterisk denotes a significant altered expression. Fold-change induction is shown in the figure. Mean ± SEM. n = 4.

BNF strongly induced CYP1A transcription in intestinal tissue of Atlantic salmon. CYP1A was 139-fold up-regulated in the MI (Fig. 4A) and 62-fold up-regulated in the DI (Fig. 5A) (t-test, P = 0.028). HSP70 was significantly up-regulated in the DI (4.8-fold, t-test, P = 0.028), and also up-regulated in the MI (3.3-fold, t-test, P = 0.058). No significant differences in transcriptional levels were observed for the GST, PCNA and caspase 6A genes (Fig. 4 and 5).

## Discussion

The intestinal mucosa provides an important site of metabolic defence against exposure to chemical contaminants, however the relationship of the intestinal integrity parameter (PCNA protein) and the toxicology parameter (CYP1A mRNA expression) is not well studied. We examined the influence of a single dose of a model toxicant (β-naphthoflavone; BNF) on the cellular localization of these two parameters together with the transcription of five genes in Atlantic salmon. Our experimental injection of BNF successfully induced a cellular stress response by strongly inducing CYP1A in both mid and distal intestine (MI and DI). The induction response of CYP1A can be quantitatively analysed by means of various molecular techniques (real-time RT-PCR, ISH) and by means of immunochemical approaches (ELISA, Western blotting).

The present study analysed intestinal mRNA CYP1A levels by real-time RT-PCR and by ISH and the real-time RT-PCR data showed that CYP1A was 139 fold (MI) and 62 fold (DI) up-regulated in the exposed animals similar to observations in the liver of the same fish [34]. The intestinal proximal distal gradient seen here is consistent with previously reported data for components belonging to the monooxygenase system [35]. The same picture was observed when CYP1A was localized within the MI tissue by ISH; exposed fish had a much higher CYP1A staining compared to the control fish (only weak and random CYP1A staining). Although the intestinal tissue is composed of several cell types, only the intestinal enterocytes gave strong CYP1A staining in the exposed group. Another interesting observation was the cellular localization of CYP1A mRNAs, CYP1A staining was always strongest at and around the nucleus where the endoplasmic reticulum (ER) is found. This observation corresponds nicely to the location of CYP1A in aquatic animals, which in many tissues is found in the ER and microsomes [36]. The pronounced CYP1A staining pattern may also indicate a quick translation into its nascent protein as CYP1A was never seen close to the apical membrane. The response pattern along the intestinal fold was common for all exposed fish and showed a characteristic strong staining in the basal area and in the apex area of the MI folds. This finding is supported by other studies on CYP1A protein induction in the intestinal epithelium [37].

Endothelial cells are the first site of interaction with blood borne toxicants and one would expect biotransformation enzymes in these cells. However, in the present study CYP1A mRNA was only localized in the enterocytes of the intestinal mucosa epithelium. Other works report a sensitive induction of CYP1A in vascular endothelia of various teleost species, reported at the protein level [38-41]. However, previous studies have focused on protein expression, which may of course explain the observed differences on cellular expression patterns. Depending on cell type, mRNA half-lives are different, ranging from only minutes to several hours, and furthermore short lived mRNA's usually encode proteins of regulatory significance such as CYP1A [42]. The cellular pattern found in the present work only gives us a snapshot of the localization of CYP1A mRNA seven days after injection of BNF. The picture would probably look different if samples were taken at different time intervals after injection. Another explanation could be that BNF accumulates in the cytoplasm of the intestinal cells and continues to re-induce CYP1A in contrast to the effect of BNF in the endothelial cells.

Several examinations have reported a clear relationship between intestinal clearance of dietary toxicants and nutrient lipids (reviewed by [21], suggesting that intestinal accumulation of BNF could be related to a lack of feed (e.g. lipids) in the present experiment. BNF accumulation in the cell cytoplasm was also suggested in a similar work looking at the spatial transcription of CYP1A in liver [34]. The route of toxicant administration is also a factor that has given a different CYP1A organ/cellular induction pattern. Dietary exposure has a much stronger effect on the intestinal mucosa compared to aqueous exposure of the toxicants [43]. Intraperitoneal injection resembles a dietary exposure as both toxicants are primarily absorbed through the portal circulation [44].

This study also investigated the effects of BNF on glutathione S-transferase (GST; belonging to the π-class) which represents one of the phase II enzymes and is part of the AhR-gene battery. In the present study BNF exposure did not have the same effect on intestinal GST transcription level as compared to the strong CYP1A response. Modest induction of GST was also seen in hepatic tissue [33]. In the present study the transcription level of GST was slightly lower (not significant) in the exposed fish compared to the control group for both MI and DI sections.

Another possible stress indicator in fish is the heat shock proteins (HSPs) and the present experimental BNF exposure induced a small effect on intestinal (MI and DI) HSP70 mRNA levels. Along with the intestinal CYP1A induction, HSP70 gave the strongest intestinal stress response out of all investigated mRNA parameters. As most of the HSP genes do not contain introns, the mRNA is rapidly translated into proteins even within minutes of exposure to the stressor [22]. Based on our study, where samples were collected as long as 7 days after exposure, it is indicated that the BNF is continuously supplied from other organs (e.g. liver) to the intestinal cells, similarly to what we indicated previously for the BNF effect observed on intestinal CYP1A. A cytoprotective role of HSP70 after stress exposure is also suggested in the present study.

One aim of this work was to investigate co-ordinated responses of BNF exposure in the intestinal tract of Atlantic salmon. In cell cultures, AhR agonists can both inhibit and enhance cell proliferation, and AhR has been shown to regulate transcription of genes involved in growth and differentiation, including proteins involved in apoptosis (reviewed by [45]. In the present study intestinal cell proliferation (PCNA) was investigated both at the transcriptional level and at the protein level. Apoptosis was investigated at the transcriptional level using one of the effector caspases, caspase 6A. Only minor and random differences were observed in PCNA and caspase 6A at the transcriptional level. There was a tendency (not significant) however, especially in the MI samples, of a lower PCNA mRNA level in the exposed fish compared to the control group. A proximal-distal proliferative gradient was seen in the control group with the MI showing a relatively higher PCNA expression compared to the distal intestine, in line with observation in the mammalian intestine [46], and similar to the metabolic zonation of the teleost intestinal tract [10,11].

Proliferating cells detected as strongly immunostained PCNA nuclei were mainly observed in the basal area of the MI folds for both exposed and control fish, but for the exposed fish a significant lower PCNA index was seen. This quantitative observation was supported visually with weak PCNA staining in the MI folds of exposed fish, indicating only a few cells being in the S-phase of the cell cycle. A similar response pattern (e.g. decrease in the intestinal proliferating cell pool) has been seen in the distal intestine of Atlantic salmon fed oxytetracycline [8] and in the MI of Atlantic salmon fed plant oils [7]. It is often seen that a down regulation of genes involved in cell proliferation (such as PCNA) often precedes morphological changes in the whole organ. This was observed in zebrafish exposed to TCDD (an AhR agonist) in which PCNA downregulation proceeded a decrease in heart growth [47,48]. Apart from the cellular localization of proliferating cells no histological evaluation on intestinal integrity was performed in the present work.

Another factor that may have affected the intestinal cell proliferation in the present study could be related to the feed restriction during the experiment. Before and after the BNF-injection fish were devoid of feed for more than 7 days and it has been observed in winter flounder, *Pseudopleuronectes americanus*, that the percentage of actively proliferating cells is substantially less in fasted fish (7 days) than in fed fish [11]. In mammalian intestine, fasting also results in reduced intestinal cell proliferation [49]. For the present investigation feeding the fish during the experimental period could have given larger effects at the transcriptional level between the exposed and control fish. It was interesting to see that the cellular immunostaining of PCNA was a more reliable and sensitive biomarker of cellular stress than PCNA mRNA levels.

In a study examining the spatial transcription of CYP1A in the liver of Atlantic salmon it was advised to always check how evenly target mRNAs are expressed in tissue sections and organs [34]. In the same study it was found that CYP1A mRNA levels were higher in the middle sections of the liver compared to the proximal and distal regions. As PCNA is not expressed in all cell types of the intestinal tract, sampling of bulk tissues (which is done for qPCR analysis) containing all cell types of the organ, usually cannot assign specific messages to particular cell types and could possible overlook differences at the cellular level.

## Conclusion

This study shows a link between the intestinal detoxification system (CYP1A mRNA) and the cell renewal system (PCNA protein) with these two processes being inversely correlated. It appears that the intestinal cells are allocating all the energy to biotransformation of BNF and to minimizing the detrimental effects of BNF by repairing damaged proteins (HSP70 mRNA expression) instead of focusing on the maintenance of the intestinal system. Finally CYP1A mRNA showed a specific intestinal cellular and tissue transcriptional pattern when localized using *in situ *hybridization (ISH). At the cellular level CYP1A mRNA was always observed at or around the cell nucleus close to the basolateral membrane and at the tissue level CYP1A mRNA was found in the basal and apex area of the intestinal fold.

## Methods

### Fish treatment

This experiment was a follow-up study from one examining liver CYP1A expression [34]. A subset of the fish used in the original experiment were selected for further study of spatial PCNA protein and CYP1A mRNA expression in intestinal cells of Atlantic salmon exposed to β-naphthoflavone (BNF). The fish treatment will only be described briefly here as more details can be found in Olsvik *et al*. [34]. All fish were starved before (24 h) and during the experiment (7 days). On 21^st ^of April 2006, 8 individuals were randomly sampled and sedated by immersion in 50 mg/L metacaine (Norsk Medisinaldepot, Oslo, Norway). Sedated fish were given an intraperitoneal injection of BNF (Sigma Chemical Co., St. Louis, MO, USA; 50 mg/kg body mass) dissolved in soybean oil (10 mg/ml) as described by Grosvik *et al. *[30]. Seven days after the exposure, 4 treated and 4 control salmon (50% of each sex) were anesthetized with metacaine and killed by a blow to the head before the post-gastric intestinal tract were dissected out. Only 4 treated and 4 control fish were chosen for further examination in the current examination, since we previously had documented the strong effect of BNF on CYP1A mRNA expression in other organs of the same fish [34] and since this mainly was a qualitative examination of PCNA activity and CYP1A expression in intestinal tissue.

### Tissue sampling

The post-gastric intestinal tract was carefully dissected out and rinsed with phosphate buffered saline (PBS) from a total of 8 Atlantic salmon with an average body weight of (n = 8, mean ± SD, 396 ± 65 g) and body length (n = 8, mean ± SD, 34.7 ± 2.7 g). Samples of mid intestine and distal intestine (MI and DI) were fixed in Bouin's solution and in phosphate-buffered formalin (4%, pH 7.2) overnight (24 h) before dehydration and paraffin embedding. Intestinal tissue for real-time RT-PCR were collected from the same intestinal samples and immediately frozen in liquefied nitrogen. Fig. 1 illustrates the intestinal sections used for tissue sampling.

### Histochemical analysis

For histochemical detection of proliferating cell nuclear antigen (PCNA) a 10 mm sample of the MI were immediately sampled and placed into Bouin's solution (15:5:1 saturated picric acid:37% formaldehyde in methanol:glacial acetic acid). After fixation, the samples were dehydrated in graded series of alcohol, equilibrated in xylene, embedded in paraffin and sectioned (5 μm) using standard histological protocols. After mounting on poly-L-lysine coated slides (Sigma-Aldrich, Oslo, Norway), sections were stained with a monoclonal antibody raised against rat PCNA using the procedure of Ortego *et al*. [16] and modified as described by Hemre *et al*. [14]. Briefly, after removing endogenous peroxidase activity with 1% H_2_O_2 _for 20 min, antigen retrieval by temperature treatment in 1% ZnSO_4 _(2 min at 780 W in microwave oven, cooling for 15 min), and protein blocking in a tris buffered saline/triton (TBS/triton) solution (50 mM Tris, 150 mM NaCl, 0.03% Triton X-100, 0.5% bovine serum albumin and 0.5% powdered milk at pH 7.6), the PCNA antibody (PC10, Chemicon, dilution 1:1000) was applied to the sections and incubated overnight at room temperature. After rinsing and protein blocking in a TBS/triton solution with 1% normal goat serum (Sigma, Poole, U.K), the sections were incubated for 1 h in biotinylated goat-anti-mouse IgG (Amersham Pharmacia Biotech, Amesham, U.K) (1:200), and finally horseradish peroxidase-conjugated avidin biotin complex (ABC complex; DAKO, Glostrup, Denmark) was applied for 30 min (1:100). The peroxidase colour reaction was started by incubating in 0.04% (w/v) diaminobenzidine (DAB; Sigma Aldrich, Oslo, Norway) Tris-HCl (50 mM, pH 7.6) after adding 0.015% H_2_O_2_. The reaction was stopped after 24 min by rinsing in tap water. PCNA-positive intestinal cells were identified as described by Ortego *et al*. and Kilemade *et al*. [16,50] and quantified according to Sanden *et al*. [5]. Six randomly selected intestinal folds were evaluated in each of three sections per fish and four fish per treatment group were examined. Only cells in the basal area of the intestinal fold and cells assumed to be in the S-phase of the cell cycle (strongly stained nuclei) were counted to avoid overestimating the proliferating cell pool [50].

### *In situ *hybridization (ISH)

Antisense and sense RNA probes were synthesized employing a non-radioactive method using a DIG RNA Labeling Kit (Roche Applied Science, Oslo, Norway). A partial cDNA [GenBank:AF364076] was subcloned into the pSPT-18 vector, and then RNA probes were transcribed *in vitro *using SP6 RNA polymerase. Paraffin blocks of MI samples (fixed overnight in phosphate-buffered formalin (4%, pH 7.2)) were stored in RNase-free sealed boxes at 4°C until cutting (5 μm), before mounted on Super Frost Plus slides (Bergman, Lillestrom, Norway) and dried overnight at 30°C. All samples were coded to ensure unknown identity until examination was finished.

The ISH protocol used in this study is slightly modified from Ebbesson *et al*. [51]. Briefly slides were rehydrated in serial concentrations of alcohol. Sections were permeabilized using proteinase K (10 μg/ml) in TrisHCl (0.05 M, pH 7.5) at RT for 5 min, before post-fixation in 4% paraformaldehyde (pH 7.5) for 10 min. Before prehybridization, slides were treated with 0.25% AA/TEA (acetic anhydride/triethanolamine) buffer (0.1 M, pH 8.0) for 10 min. Slides where then prehybridized in 10% dextran sulphate, 5 × SSC (SDS sodium citrate buffer), 50% formamide, 5× Denhardts, 250 μg/ml tRNA, 500 μg salmon sperm DNA for 2 h at RT in a moist chamber. Slides were then incubated with the DIG-AP cRNA probe (500 ng/ml hyb buffer) in a moist chamber for 16 h at 67°C. After the hybridization, slides were washed using the following protocol: 5 × SSC at RT for 30 min, 30% formamide in 5 × SSC at 67°C for 15 min, cooling to RT, before a final wash in 0.2 × SSC at RT for 5 min. To control the trueness of the hybridization and the probes, a brief RNase treatment was performed. Probes were then visualized by incubating the slides with a sheep anti-DIG (1:1500 in blocking solution; heat inactivated goat serum) solution at 8°C over night. The alkaline phosphatase reaction was performed by incubating the slides in reaction solution NBT/BCIP (Nitro-Blue Tetrazolium Chloride/5-Bromo-4-Chloro-3'-Indolyphosphate p-Toluidine Salt) + Levamizole 0.3 mg/ml) for 1.5 h at RT. The colour reaction was stopped in a stop solution (10 mM TrisHCl, 1 mM EDTA, 0.9% NaCl, pH 8.0). Sections were counterstained in methyl green (Sigma Chemical Co., St. Louis, MO, USA) for 7 min prior to quick dehydration, cleared in xylene, and mounted in entellan.

Sections stained for PCNA and CYP1A mRNA were examined with an Olympus BX51 light microscope (Olympus Hamburg, Germany) and micrographs obtained using an Olympus DP50 digital imaging system mounted on the microscope. Histological slides were qualitatively evaluated using the software cell*B 2006 (Olympus, Hamburg, Germany).

### RNA extraction, RNA quality and integrity

Tissues were thoroughly homogenized before RNA extraction with zirconium beads (4 mm) in a Retsch MM 301 homogenizer (Retsch GmbH, Haan, Germany). Total RNA was extracted using Trizol reagent (Invitrogen, Life Technologies, Carlsbad, CA, USA), according to the manufacturer's instructions and stored in 100 μl RNase-free MilliQ H_2_O. Genomic DNA was eliminated from the samples by DNase treatment according to the manufacturer's description (DNA-*free*, Ambion, Austin, TX, USA). The RNA was then stored at -80°C before further processing. The quality of the RNA was assessed with the NanoDrop^® ^ND-1000 UV-Vis Spectrophotometer (NanoDrop Technologies, Wilmington, DE, USA) and the Agilent 2100 Bioanalyzer (Agilent Technologies, Palo Alto, CA, USA). The RNA 6000 Nano LabChip^® ^kit (Agilent Technologies, Palo Alto, CA, USA) was used to evaluate the integrity of the RNA. All samples had a RNA integrity number (RIN) of 7.0 or higher (n = 16, mean ± SD, 8.7 ± 0.7).

### Real-time quantitative RT-PCR

PCR primer sequences used for quantification of the genes encoding β-actin, elongation factor 1 alpha (EF1A_B_), acidic ribosomal protein (ARP), CYP1A, GST π, PCNA, HSP70 and caspase 6A are shown in Table 1. For more detailed information on the PCR assays, see Olsvik *et al*. [7,34]. The primer pairs amplified PCR products between 59–121 basepairs (bp) long. The *geNorm *software was used to evaluate the stabilities of the three reference genes. A normalization factor based on all three examined reference genes (β-actin, EF1A_B _and ARP) was used to calculate mean normalized expression (MNE) of the target genes [52]

**Table 1:** PCR primers and amplicon sizes of the reference and target genes used in the present study.

***Gene***	***Forward primer 5'-3'***	***Reverse primer 5'-3'***	***Product size (bp*)**
Beta-actin	CCAAAGCCAACAGGGAGAA	AGGGACAACACTGCCTGGAT	92
EF1AB	TGCCCCTCCAGGATGTCTAC	CACGGCCCACAGGTACTG	59
ARP	TCATCCAATTGCTGGATGACTATC	CTTCCCACGCAAGGACAGA	101
CYP1A	TGGAGATCTTCCGGCACTCT	CAGGTGTCCTTGGGAATGGA	101
GST	ATTTTGGGACGGGCTGACA	CCTGGTGCTCTGCTCCAGTT	81
PCNA	CGTGGAGAGTATGGATTCGTCCCACG	CGCAGCGGTAAGAGTCGAAC	80
HSP70	CCCCTGTCCCTGGGTATTG	CACCAGGCTGGTTGTCTGAGT	121
Caspase 6A	TGAGCCACGGAGAGAACGA	CCCACCAGGCTCTTACACTTG	103

### Statistics

A simple t-test was used to compare differences in PCNA and apoptosis indices between the two groups of Atlantic salmon using the software Statistica TM, Release 4.5 (Statsoft, Inc., Tulsa, OK, USA 1993). The t-test was also used to analyze gene expression differences between the control and the BNF-exposed fish. A significance level of P < 0.05 was used for all tests.

## Authors' contributions

MS initiated the research, evaluated all the *in situ *hybridization, immunohistochemistry and histology work and also wrote the paper. PAO was responsible for the exposure experiment and did the quantitative real-time RT-PCR analysis and helped drafting those parts of the manuscript. Both authors read and approved the final manuscript.
